# Supplementary material for: Bridging the Knowledge Gap: A National Survey on MASLD Awareness and Management Barriers in the Saudi Population
Source: Healthcare (Basel). 2025 Dec 18;13(24):3322. doi: 10.3390/healthcare13243322 (PMC12732865; doi:10.3390/healthcare13243322)
Supplement: Supplementary file 1 [file healthcare-13-03322-s001.zip › healthcare-4004672-supplementary.pdf]

Table S1. Demographics of participants.

Variable	n (%)
Gender	
Male	231(46)
Female	271(54)
Age	
18-24	257(51.2)
25-33	53(10.6)
34-51	122(24.3)
52-64	57(11.4)
> 65	9(1.8)
Job	
Employer	172(34.3)
Unemployed	45(9.0)
Students	240(47.8)
Retired	45(9.0)
Education level	
High school	88(17.5)
University	384(76.3)
Postgraduate	30(6.0)
body index	
below normal	36(7.2)
Normal	230(45.8)
Overweight	148(29.5)
Obesity	34(6.8)
I don't know	54(10.8)

Table S2. Participants' knowledge regarding NAFLD

Variables	n (%)
Do you think you can get fatty liver without drinking alcohol?	226 (45)
	69 (13.7)
Yes	207 (41.2)
No	
I don't know	
Have you ever heard of the term "non-alcoholic fatty liver disease" or "fatty liver"	
Yes	237 (47.2)
No	265 (52.8)
Do you think non-alcoholic fatty liver disease is a disease that requires hospital treatment?	
Agree	300 (59.8)

Disagree<br>I don't know	27 (5.4)<br>175 (34.9)
NFLD is life-threatening<br>Yes<br>No<br>I don't know	 255 (50.8)<br>34 (6.8)<br>212 (42.2)
Methods for diagnosis<br>Body mass index<br>Ultra sound<br>Blood test<br>All the above<br>I don't know	 40 (8)<br>40 (8)<br>49 (9.8)<br>236 (47.6)<br>133 (26.5)

Table S3. Knowledge of participants about management of NAFLD.

**Variables**	n (%)
Change the diet (reduce sugars, processed foods, and saturated fats)	333 (66.3)
Physical activity	326 (64.9)
Controlling blood sugar levels for patients with diabetes, using medications if necessary	171 (34.3)
Vitamins	155 (30.9)
Lowering cholesterol and triglyceride levels through diet and medication if necessary	275 (54.8)
Eat a balanced diet rich in vegetables, fruits, and healthy proteins, with an emphasis on fiber and whole grains.	305 (60.8)

Table S4. Association between participants' knowledge levels and their demographics.

Variables	Knowledge levels				95% Confidence Interval (CI)	p-value
	Good n(%)	Fair n(%)	Poor n(%)	Total		
			Gender			
Male	61(26.4)	91(39.4)	79(34.2)	231	0.2 to 0.4	0.514
Female	64(23.6)	101(37.3)	106(39.1)	271	0.18 to 0.4	
			Age			
18-24	42(16.3)	108(42.0)	107(41.6)	257	0.1 to 0.5	0.002*
25-33	17(29.8)	20(35.1)	20(35.1)	57	0.2 to 0.5	
34-51	43(35.2)	38(31.1)	41(33.6)	122	0.2 to 0.4	
52-64	21(36.8)	21(36.8)	15(26.3)	57	0.1 to 0.6	
65 and above	2(22.2)	5(55.6)	2(22.2)	9	0.03 to 1.3	
			Job			
Employer	58(33.7)	55(32.0)	59(34.3)	172	0.24 to 0.44	0.001*
Unemployed	14(31.1)	18(40.0)	13(28.9)	45	0.15 to 0.63	
Students	38(15.8)	99(41.3)	103(42.9)	240	0.11 to 0.52	
Retired	15(33.3)	20(44.4)	10(22.2)	45	0.11 to 0.69	
		Education level				
High school	23(26.1)	37(42.0)	29(31.8)	88	0.16to 0.58	0.008*

University	87(22.7)	145(37.8)	152(39.6)	384	0.18 to 0.46	
Postgraduate	15(50.0)	10(33.3)	5(16.7)	30	0.05 to 0.82	
Body mass index						
Below normal	5(13.9)	15(41.7)	16(44.4)	36	0.05 to 0.72	
Normal	52(22.6)	88(38.3)	90(39.1)	230	0.16 to 0.48	0.230
Overweight	45(30.4)	56(37.8)	47(31.8)	148	0.22 to 0.49	
Obesity	13(38.2)	11(32.4)	10(29.4)	34	0.14 to 0.58	
I don't know	10(18.5)	22(40.7)	22(40.7)	54	0.09 to 0.62	

Table S5. Participant's attitude towards NAFLD.

**Variables**	**Yes** n (%)	**No** n (%)
Do you believe that obesity causes NAFLD	243(48.4)	259(51.6)
Do you believe that NAFLD is caused by diabetes?	146 (29.1)	356 (70.9)
Do you believe that hypertension affects NAFLD?	120(23.9)	382(76.1)
Do you believe that liver cancer can be caused by NAFLD?	215 (42.8)	287(57.2)
Do you believe that high blood cholesterol cause NAFLD?	260(51.8)	242(48.2)
Do you think that NAFLD can cause cardiovascular diseases	149(29.7)	353(70.3)

Table S6. Association between Participants' attitude levels and their demographics.

**Variables**	Knowledge levels				95% Confidence Interval (CI)	p-value
	Good n(%)	Fair n(%)	Poor n(%)	Total		
Gender						
Male	64(27.7)	83(35.9)	84(36.4)	231	0.23 to 0.45	0.520
Female	68(25.1)	91(33.6)	112(41.3)	271	0.19 to 0.49	
Age						
18-24	72(28.0)	82(31.9)	103(40.1)	257	0.22 to 0.49	0.614
25-33	12(21.1)	23(40.4)	22(38.6)	57	0.11 to 0.61	
34-51	26(21.3)	47(38.5)	49(40.2)	122	0.14 to 0.51	
52-64	19(33.3)	18(31.6)	20(35.1)	57	0.18 to 0.54	
65 and above	3(33.3)	4(44.4)	2(22.2)	9	0.07 to 1.1	
Job						
Employer	43(25.0)	62(36.0)	67(39.0)	172	0.18 to 0.49	0.725
Unemployed	9(20.0)	17(37.8)	19(42.2)	45	0.09 to 0.66	
Students	70(29.2)	76(31.7)	94(39.2)	240	0.23 to 0.47	
Retired	10(22.2)	19(42.2)	16(35.6)	45	0.11 to 0.58	
Education level						
High school	26(29.5)	33(37.5)	29(33.0)	88	0.19 to 52	0.261
University	94(24.5)	132(34.4)	158(41.1)	384	0.19 to 0.48	
Postgraduate	12(40.0)	9(30.0)	9(30.0)	30	0.13 to 0.69	
Body mass index						

Under normal	8(22.2)	8(22.2)	20(55.6)	36	0.01 to 0.85	0.084
Normal	62(27.0)	73(31.7)	95(41.3)	230	0.21 to 0.51	
Overweight	43(29.1)	57(38.5)	48(32.4)	148	0.21 to 0.49	
Obesity	11(32.4)	14(41.2)	9(26.5)	34	0.12 to 0.69	
I don’t know	8(14.8)	22(40.7)	24(44.4)	54	0.06 to 0.66	

Table S7. Participants’ responses to the management status and obstacles to NAFLD management.

**Variables**	n (%)
Diagnosis with NAFLD	
Yes	39 (7.8)
No	453 (92.2)
During your hospital visit, have you been recommended to change your lifestyle?	
Yes	
No	29 (74.4)
	10 (25.6)
Did you visit the hospital for more tests and management of NAFLD?	
Yes (go to the prevention/management question)	
No (go to the reason question)	24 (61.5)
	15 (38.5)
Your reason for not following-up with another hospital visit? (Multiple answers allowed) (n=15)	
Not considered fatty liver a serious disease	5 (33.3)
I believed that by changing my lifestyle on my own, I could control the illness (weight management, exercise management, etc.)	7 (46.7)
Lack of time to visit the hospital	
The cost of medical care	2 (13.3)
My doctor has never advised me that I require illness management.	4 (26.7)
	5 (46.7)
Prevention/management of NAFLD (n=24)	
I am not managing my NAFLD in any specific way.	8 (33.3)
Supplements for hyperlipidemia and the liver are available at pharmacies or online.	5 (20.8)
Hospital-prescribed drugs for hyperlipidemia and liver	6 (25)
Reduction in calorie intake	5 (20.8)
Increase in the amount of exercise	7 (29.2)
Weight loss	10 (41.7)

Table S8. Perception of participants about the most important for the effective long-term management of non-alcoholic fatty liver disease.

Variables	n (%)
What do you think is the most important aspect of managing long-term non-alcoholic fatty liver disease? (Multiple answers)	
Make time for lifestyle changes	320 (63.7)
Health with treatment costs	221 (44)
Providing nutritional advice and periodic management by a nutritionist	286 (57)
Providing advice on how to exercise and periodic management by a sports specialist	232 (46.2)
Instructions on proper diet and exercise provided by a physician	
	283 (56.4)
If there is a mobile app for the prevention or management of non-alcoholic fatty liver disease, would you be willing to participate?	
Strongly agree	
Agree	172 (34.3)
Neutral	47 (9.4)
Disagree	122 (24.3)
Strongly disagree	61 (12.2)
If there is a program to visit public health centers to prevent or manage non-alcoholic	100 (19.9)
fatty liver disease, would you be willing to participate?	
Willing to actively participate	
Willing to participate	172 (34.3)
Neutral	47 (9.4)
Little interest in participating	122 (24.3)
No interest in participating	61 (12.2)
	100 (19.9)
